# A computational analysis of protein-protein interaction networks in neurodegenerative diseases

**DOI:** 10.1186/1752-0509-2-52

**Published:** 2008-06-20

**Authors:** Joaquín Goñi, Francisco J Esteban, Nieves Vélez de Mendizábal, Jorge Sepulcre, Sergio Ardanza-Trevijano, Ion Agirrezabal, Pablo Villoslada

**Affiliations:** 1 Neuroimmunology laboratory, Department of Neuroscience, Center for Applied Medical Research, University of Navarra, Spain; 2 Department of Physics and Applied Mathematics, University of Navarra, Spain; 3 Systems Biology Unit, Department of Experimental Biology, University of Jaen, Spain; 4 Department of Computational Sciences and Artificial Intelligence, University of the Basque Country, San Sebastian, Spain

## Abstract

**Background:**

Recent developments have meant that network theory is making an important contribution to the topological study of biological networks, such as protein-protein interaction (PPI) networks. The identification of differentially expressed genes in DNA array experiments is a source of information regarding the molecular pathways involved in disease. Thus, considering PPI analysis and gene expression studies together may provide a better understanding of multifactorial neurodegenerative diseases such as Multiple Sclerosis (MS) and Alzheimer disease (AD). The aim of this study was to assess whether the parameters of degree and betweenness, two fundamental measures in network theory, are properties that differentiate between implicated (seed-proteins) and non-implicated nodes (neighbors) in MS and AD. We used experimentally validated PPI information to obtain the neighbors for each seed group and we studied these parameters in four networks: MS-blood network; MS-brain network; AD-blood network; and AD-brain network.

**Results:**

Specific features of seed-proteins were revealed, whereby they displayed a lower average degree in both diseases and tissues, and a higher betweenness in AD-brain and MS-blood networks. Additionally, the heterogeneity of the processes involved indicate that these findings are not pathway specific but rather that they are spread over different pathways.

**Conclusion:**

Our findings show differential centrality properties of proteins whose gene expression is impaired in neurodegenerative diseases.

## Background

The structural and functional relationships underlying the organization of living systems imply the need to coordinate molecular interactions, principally those involving gene expression and protein activity. Although the genome in each cell of a given organism is virtually the same, dynamic changes in gene expression and thus in the protein content depend on the functional state of the cell [1]. Genome-wide expression profiles using DNA arrays, together with the development of bioinformatic approaches [2], enable both genetic and protein-protein interaction (PPI) networks to be modeled, thereby helping to understand how biological networks operate [3].

From a systems point of view, the arrangement of molecular networks from gene expression data based on known interactions permits the understanding of the basic mechanisms upon which the complexity and adaptability of a living cell is founded [4]. This information also helps to decipher processes involved in illness, for instance the molecular heterogeneity of cancer [5]. However, and consistent with the model of multifactorial diseases, it is difficult to find genes that account for direct genotype-phenotype correlations [6]. Thus, network modeling and topological analysis at the meso-scale (intermediate level between local and global features of networks) may provide additional knowledge about the particular properties of genes and proteins involved in diseases of multifactorial nature, where the pathogenesis does not depend on the malfunction of a single gene or protein [7]. In this case, the analysis of gene, protein and pathway interactions might indicate common properties of good candidates to be targeted by therapy. In addition, understanding the emergent properties of a system might help identify new targets that would not be captured by a molecular approach [8].

Multiple Sclerosis (MS) is a chronic inflammatory and neurodegenerative disease of the central nervous system (CNS) [9]. Although its etiology remains elusive, the interplay between environmental and genetic factors is ultimately thought to be critical for the development of the disease. MS is considered as an autoimmune disease due to the presence of inflammatory infiltrates in the brain in the absence of infection, and through its association with HLA alleles, among other factors [10]. The chronic inflammatory activity within the CNS is the main mediator of tissue damage, even in the late neurodegenerative stage of the disease that involves widespread demyelination and axon loss [11]. In addition to the autoimmune processes, MS also has a neurodegenerative component whereby axons and neurons are lost through unknown processes in the late chronic stages of the disease. Several lines of evidence suggest that the degeneration of demyelinated axons is the most important factor in MS neurodegeneration [12]. Thus, MS is a multifactorial disease in which many immune system and CNS pathways are involved [13]. Current therapies partially ameliorate the inflammatory process, but more effective therapeutic approaches are required to stop disease progression and prevent neurodegeneration.

Alzheimer's Disease (AD) is the most common neurodegenerative disease and it represents one of the biggest unmet needs in modern medicine. AD is characterized by the loss of neurons in conjunction with the presence of oxidative stress, axonal dystrophy, mature senile plaques and neurofibrillary tangles [14]. A set of gene mutations involved in the amyloid beta and tau pathways have been associated with hereditary AD and, in conjunction with neuropathological findings, it has been demonstrated that amyloid and tau are involved in the pathogenesis of AD. However, current evidence suggests that sporadic AD is a multifactorial disease in which many pathways are involved [15,16]. Indeed, recent studies have also identified molecular abnormalities in the blood of patients with AD [17]. Because the AD therapies available are symptomatic, and considering the epidemic proportions of this disease in western countries, the development of new therapies to stop its progress is an important health priority.

To better understand the basis of neurodegenerative diseases, we set out to study the centrality related features of proteins whose genes were differentially expressed (seed proteins) in MS and AD with respect to their protein neighbors. The main features examined were the degree and the betweenness of these seed proteins and its comparison to their neighbors.

## Results

**Figure 1:**
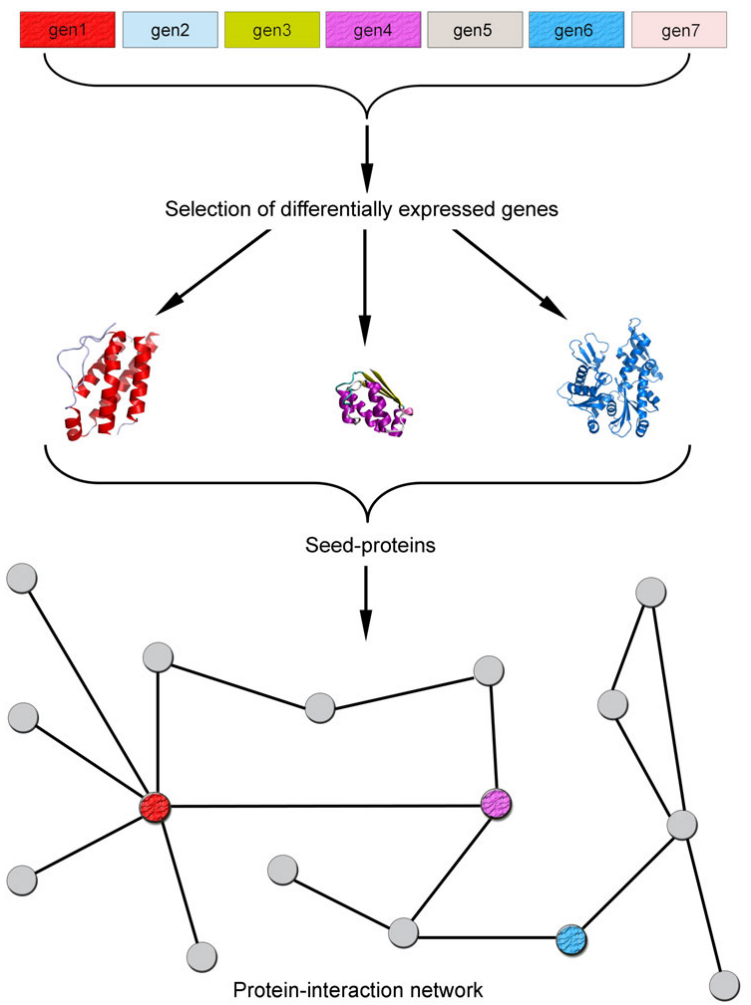
**Retrieval and representation of each disease network.** The differentially expressed genes in MS or AD using blood or brain tissue were obtained from published DNA array studies. The corresponding protein (seed-protein) for each differentially expressed gene was identified in public databases (STRING). The network in which such proteins were embedded was built by retrieving the first neighbor of each protein in the protein-protein interaction database available at the STRING database.

The four networks studied here were obtained as indicated in figure 1, whereby seed-proteins were identified from DNA array studies on MS and AD, both in blood and CNS tissue, and the interacting neighbors were derived from an analysis of the STRING database [18].

**Figure 2:**
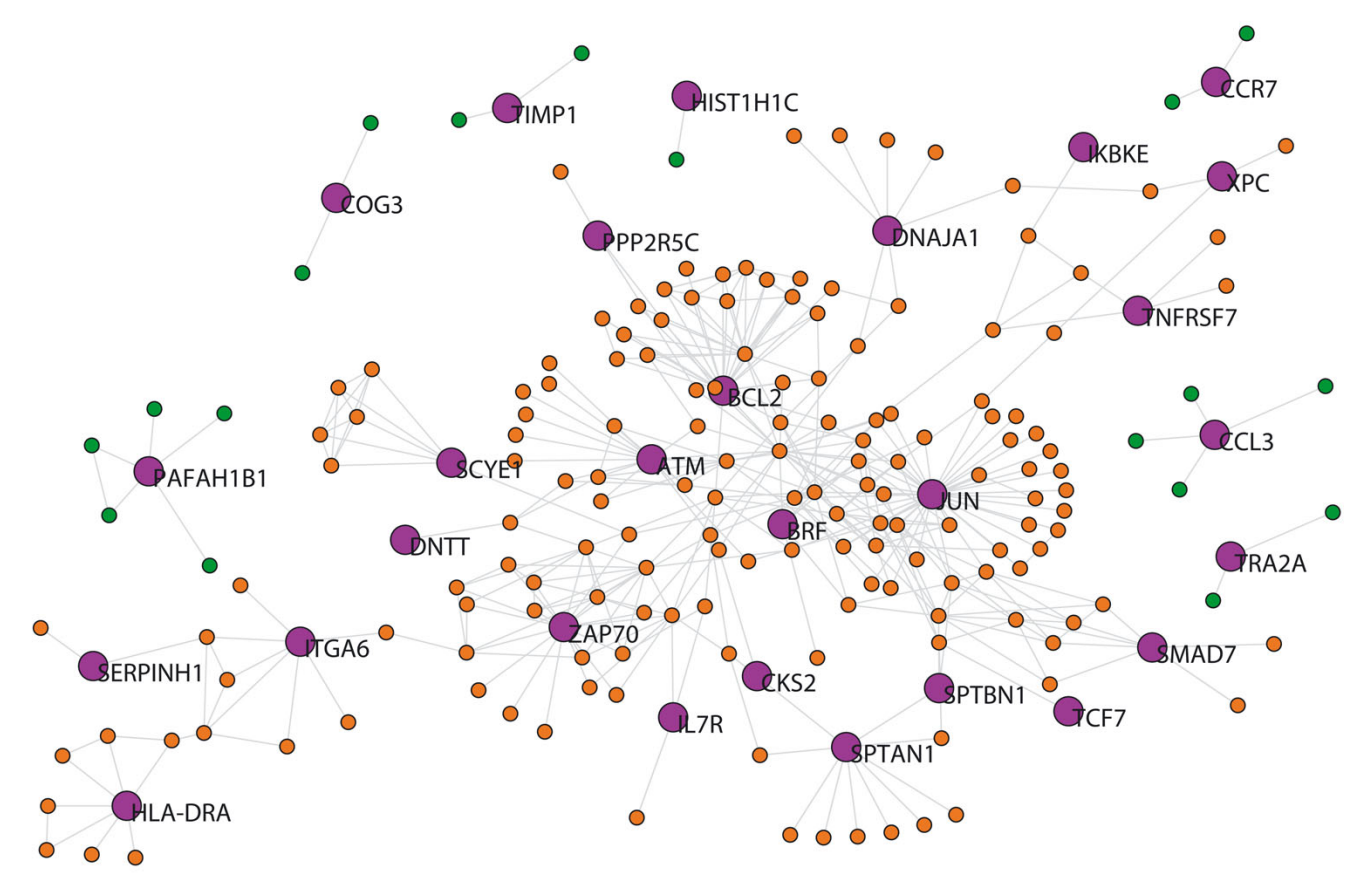
**MS-blood network.** Purple nodes indicate the seed-proteins with their name. Orange nodes indicate neighboring proteins belonging to the giant component. Green nodes indicate neighbors that do not belong to the giant component. The graphs were built using Pajek software and the network files are available as .net files from the authors upon request.

The MS network from blood tissue (MS-blood) contains 28 out of the 42 seed-proteins and 177 neighbors were derived. The 14 seed-proteins that had no links (i.e. there was no experimental evidence of interactions) were not included in the network analysis described in Table 1. The giant component of this network has 180 proteins. Accordingly, we studied the measurements listed in Table 1 in a network with 205 nodes (Fig. 2): number of nodes (*N*), average degree (<*k*>), clustering coefficient (<*C*>), diameter (*D*) and mean shortest path length (*mspl*). The differences in the average degree and the betweenness distribution between the seed-proteins and their neighbors are shown in Table 2. The seed-proteins of the MS-blood network have a lower average degree and betweenness than neighbors (p < 0.05 in both cases; Table 2). We assessed whether some of the functional pathways (identified by Gene Ontology (GO)) were overrepresented in the gene set corresponding to MS seed-proteins. We found that 36 GO terms were overrepresented in seed proteins after false discovery rate (FDR) correction [see additional file 1]. Such pathways not only included terms related with the activity of the immune system but also with many other cellular process, such as metabolic process, protein degradation and the response to stress.

**Table 1:** Network measurements for the four disease networks.

		**MS-blood**		**AD-blood**		**MS-brain**		**AD-brain**	
		
**Symbol**	**Description**	**full**	**giant comp**	**full**	**giant comp**	**full**	**giant comp**	**full**	**giant comp**
N	number of nodes	205	180	96	82	148	109	134	84
<k>	average degree	3.77	4.08	5.1	5.63	3.12	3.59	2.85	3.31
<C>	clustering coefficient	0.32	0.35	0.43	0.44	0.26	0.29	0.32	0.35
D	diameter	-	14	-	12	-	13	-	11
mspl	mean shortest path length	-	4.76	-	5.5	-	4.6	-	5.41

The number of nodes, average degree (<k>), clustering coefficient (<C>), diameter of the network (D) and mean shortest path length (mspl) are shown for the overall networks (full) or for the giant component of each network (by considering only the nodes linked to the bigger subnetwork). The four networks analyzed are the MS in blood tissue (MS-blood); AD in blood tissue (AD-blood), MS in brain tissue (MS-brain) and the AD in brain tissue (AD-brain).

**Table 2:** Connectivity analysis of the disease networks.

	**Degree<k>**	**Non-zero Degree**	**Betweenness**
**ADBlood**	<0.05^1^	<0.05^1^	0.75
**ADBrain**	<0.05^1^	<0.05^2^	<0.05^2^
**MSBlood**	<0.05^1^	0.16	<0.05^2^
**MSBrain**	<0.05^1^	0.4	0.64

Results are displayed as the p value of the Kolmogorov-Smirnov test for the four networks analyzed: the MS in blood tissue (MS-blood); AD in blood tissue (AD-blood), MS in brain tissue (MS-brain) and the AD in brain tissue (AD-brain). Non-zero degree and betweenness were calculated after excluding the non-connected (non-zero) nodes.

^1 ^Seed proteins significantly smaller; ^2 ^seed-proteins significantly higher

**Figure 3:**
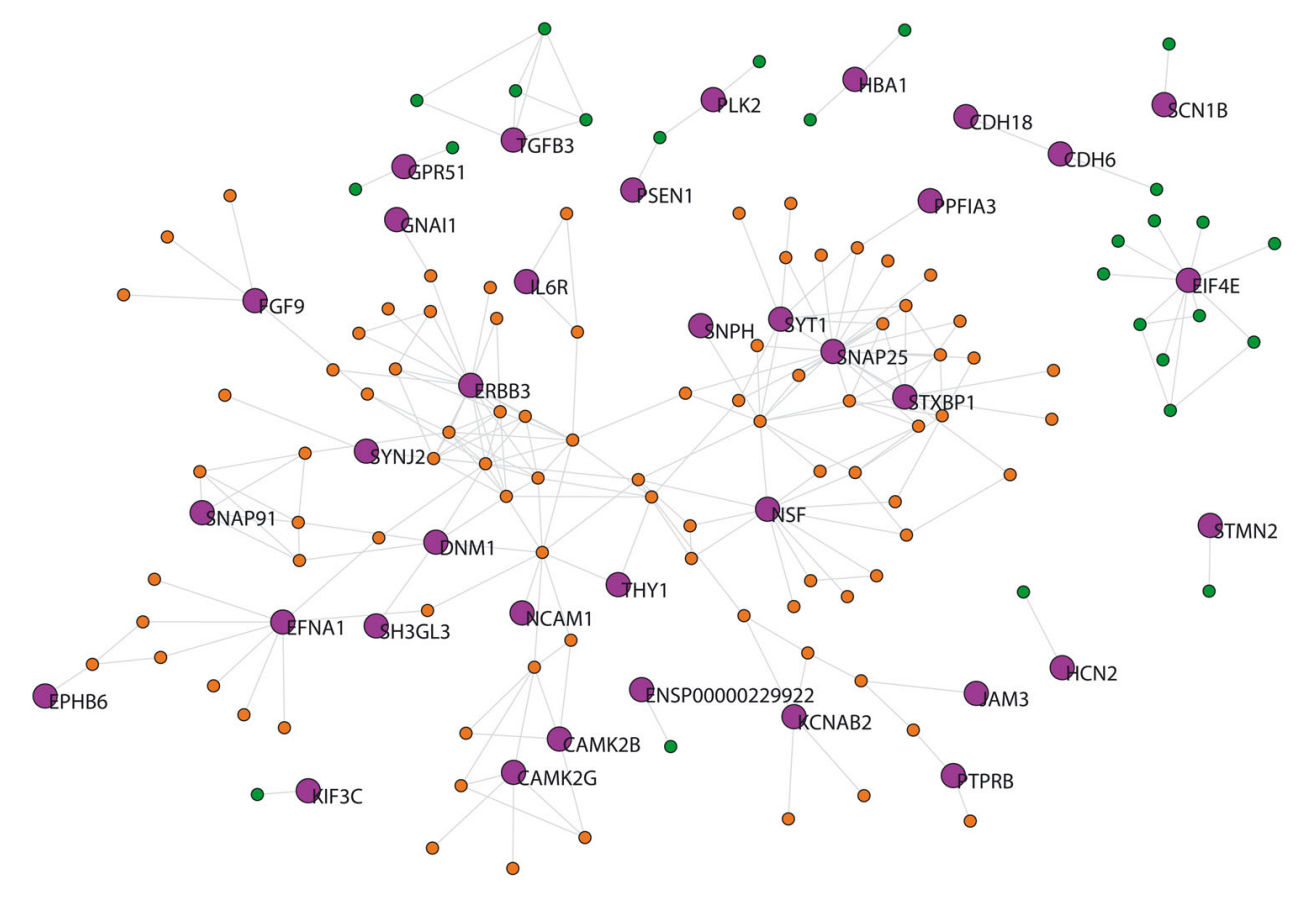
**MS-brain network.** Purple nodes indicate the seed-proteins with their name. Orange nodes indicate neighbors proteins belonging to the giant component. Green nodes indicate neighbors that are not included in the giant component.

The MS network from brain tissue (MS-brain) contains 38 out of 99 seed-proteins (61 seed-proteins had no links) and 96 neighbors. Thus the network has 134 nodes and its giant component has 109 proteins (Fig. 3). The seed-proteins of the MS-brain network have a lower average degree than the neighbor proteins (Table 2), and we found 67 GO terms overrepresented in seed proteins after FDR correction [see additional file 2]. Again, overrepresented pathways not only included components of the immune response but also those involved in synaptic transmission, neurogenesis and neuron differentiation, among others.

**Figure 4:**
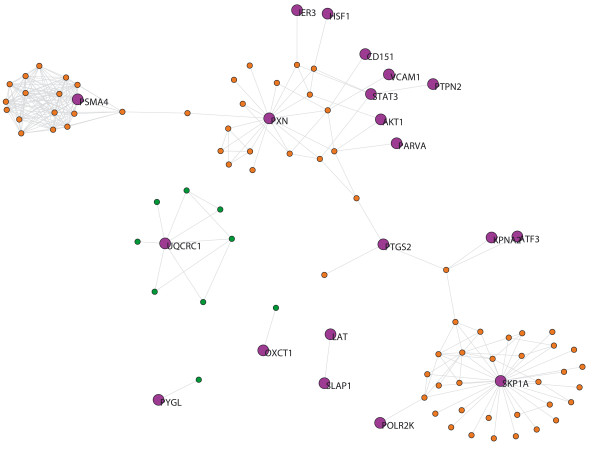
**AD-blood network.** Purple nodes indicate the seed-proteins with their name. Orange nodes indicate neighbors proteins belonging to the giant component. Green nodes indicate neighbors that are not included in the giant component.

The AD network from blood tissue (AD-blood) contains 20 out of 142 seed-proteins (122 seed-proteins had no links) and 76 neighbors. Thus the network has 96 nodes and its giant component has 82 proteins (Fig. 4). The seed-proteins of the AD-blood network have a lower average degree than their neighbor proteins (Table 2) and we found no GO terms overrepresented in seed proteins when compared to their neighbors after FDR correction [see additional file 3].

**Figure 5:**
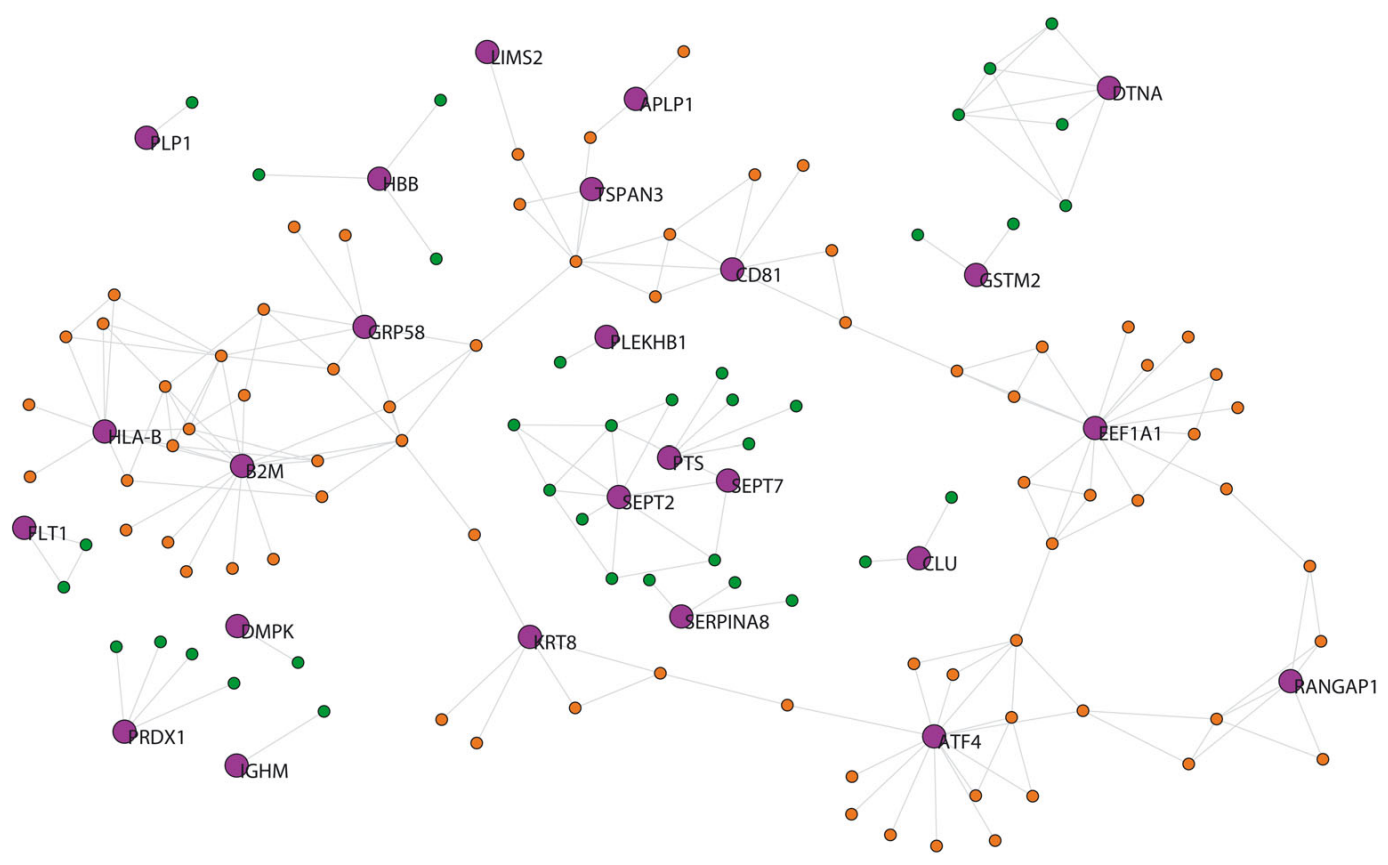
**AD-brain network.** Purple nodes indicate the seed-proteins with their name. Orange nodes indicate neighbors proteins belonging to the giant component. Green nodes indicate neighbors that are not included in the giant component.

The AD network from brain tissue (AD-brain) contains 25 out of 35 seed-proteins (10 seed-proteins had no links) and 109 neighbors. Thus the network has 134 nodes and its giant component has 84 proteins (Fig. 5). The seed-proteins of the AD-brain network have a lower average degree and betweenness than their neighbor proteins (Table 2). We found 18 GO terms that were overrepresented in seed proteins after FDR correction [see additional file 4], terms that were involved in CNS development, oxygen transport or complement activation, among others.

As indicated in Tables 1 and 2, we found seed-proteins displayed a lower average degree with respect to the degree of their PPI neighbors in both diseases and in both tissues. In addition, direct interactions between seed proteins were very low: MS-blood: 1 (total links: 386); MS-brain: 4 (total links: 231); AD-blood: 2 (total links: 245); AD-brain: 2 (total links: 191). There were not big differences in the network measurements (<*k*>, <*C*>, *D *and *mspl*) among the four disease networks studied, indicating certain homogeneity in the architecture of the PPI subnetworks analyzed in this study (Table 1). With regards the centrality of seed proteins, our study shows a low correspondence between their degree and betweenness (Table 2), indicating that critical proteins in disease pathogenesis are not highly connected, but tend to be located in bottleneck regions.

## Discussion

Network theory provides a useful tool to study the complexity of neurodegenerative diseases. Here we report a novel approach to study PPI networks at the meso-scale based on the products of genes differentially expressed in MS and AD. Our approach was to analyze PPI networks based on seed-protein neighborhoods from the genes that were differentially expressed in DNA array studies. The method for growing networks from seed-proteins is critical for determining their topological properties [19]. For this reason, the network growth in our study was carried out by expanding it through experimentally validated protein interactions. The stability, dynamics and functioning of networks are generally characterized by determining the topology of the map, i.e., the configuration of its nodes and the connecting edges [20]. For example, networks with a scale-free topology are resistant to random failure but they are vulnerable to targeted attack, specifically against the most connected nodes. In terms of identifying common properties among the genes involved in neurodegenerative disorders, very interesting results were obtained by carrying out a topological analysis. There were multiple pathways affected by proteins with a low degree, and half the time with high betweenness.

During the last decade, network studies have been applied to biological data bearing in mind that the degree of connectivity is a key property of any network, as demonstrated in yeast [21]. The most common approach to identify key nodes consists of obtaining networks from high throughput data and having obtained the network, searching for the most connected nodes (hubs). The underlying assumption was that these hubs could be critical to explain the pathogenesis of diseases. However, betweenness is another key indicator of centrality that demonstrates how nodes with a low degree of centrality may be relevant in a network (i.e. bottleneck effect) [22,23]. Our study was performed from a novel viewpoint, since we analyzed whether degree was any different respect to the PPI neighbors starting from critical nodes (in terms of differentially expressed genes). Accordingly, we found that the degree of seed-proteins was lower than that of the PPI neighbors, situating seed proteins in peripheral regions of the network. According to our results of the GO analysis, such peripheral regions are distributed among several pathways that could be involved in disease. Indeed, our results are in agreement with a recent study in asthma showing that hubs exhibit small changes in gene expression [24]. Therefore our results support the application of strategies other than those previously applied, whereby only hubs that might compromise the robustness of networks were generally searched [25,26].

The fact that we obtained similar results with regards the low average degree of seed proteins in two diseases and two different tissues suggests that this might be a common property in complex diseases, more relevant than the issues associated with the techniques applied such as DNA array technology. However, our approach relies on the current knowledge of interactions, which depends more on how much the gene/protein has been studied than on how many real interactions it participates in. Although it is more difficult to relate gene expression data from hubs with that of other genes, this would not bias our analysis since we focused on whether genes that are differentially expressed (but not necessarily correlated) have a particular distribution with regards their neighbors (neighbors found in a database that includes structural and experimental evidence and not correlation profiles).

We can consider complex diseases as an evolutionary stage in which the pathogenesis process hijacks the robustness of the biological pathways. Such an event may be followed by a cascade of failures in these pathways [8,27]. In this sense and from a therapeutic point of view, it may be necessary to target many of the pathways involved following a systems biology rationale, and based on the dynamics and topology of the networks involved. The aim of this therapy would be to drive those pathways to a non-pathological state or at least, to a less deleterious state.

The topological implications of the observed scale-free properties in biological networks would indicate that the best therapeutic targets to modify network behavior would be the genes (or proteins) corresponding to the hubs in the network. However, our findings suggest that less extensively connected proteins might be more appropriate therapeutic targets than hyper-connected ones, at least in neurodegenerative diseases. The fact that in both diseases (MS and AD) and in two different tissues analyzed (blood and cerebral tissue), seed-proteins are weakly connected nodes taking part in many different pathways, strengthens the concept of the multifactorial pathogenesis of neurodegenerative diseases. Thus, our results suggest that to modify the disease course we need to target many genes or proteins in several pathways. In a previous network analysis in MS we demonstrated that therapies act on different regions of the gene network that control T-cell activation, suggesting that a pleiotropic activity is required in order to modulate the immune response [28]. In addition, recent network studies in neurodegenerative diseases suggest that several common pathways are involved in their pathogenesis, reinforcing the need to interact with several regions of the PPI network [29,30]. Another reason why hubs might not be good therapeutic targets is because their critical role in the network modules might prevent them from fluctuating substantially. For the same reason, we can speculate that networks would poorly tolerate modifications in hub behavior without spreading such changes across the network and thereby, inducing significant side effects.

The results we present here indicate that both neurodegenerative diseases (MS and AD) share common characteristics, such as the low degree of seed-proteins and in two of the four disease networks, a high degree of betweenness. These findings mainly situate seed-proteins in peripheral regions of the PPI map (in terms of degree), involved in different pathways as indicated by the associated GO terms and the direct interactions, and integrated into subnetworks of the complete Human proteome network.

## Methods

### Definitions

Some definitions are introduced to better explain the development of our topological studies:

- *Seed-proteins*: proteins whose genes were differentially expressed in DNA array studies focused on the specific disease and on a particular tissue. In this study, the diseases considered are Multiple Sclerosis (MS) and Alzheimer Disease (AD) and the tissues are blood and brain.

-*MS-blood seed-proteins*: proteins whose genes were differentially expressed in DNA array studies of blood from MS patients [31].

-*MS-blood neighbors*: nodes selected as a consequence of adding experimentally validated interactions starting from seed-proteins.

-*MS-blood network*: network that includes MS seed-proteins, MS-neighbors and their interactions. Only seed-proteins linked to neighboring proteins were included in the network analysis (isolated seed-proteins were not included in the analysis shown in Table 1).

-*MS-brain seed-proteins*: proteins whose genes were differentially expressed in DNA array studies of brain tissue from MS patients [32].

-*MS-brain neighbors*: nodes selected as a consequence of adding experimentally validated interactions starting from seed-proteins.

-*MS-brain network*: network that includes MS seed-proteins, MS-neighbors and their interactions.

-*AD-blood seed-proteins*: proteins whose genes were differentially expressed in DNA array studies of blood from AD patients [33].

-*AD-blood neighbors*: nodes selected as a consequence of adding experimentally validated interactions starting from AD seed-proteins.

-*AD-blood network*: network that includes AD seed-proteins, AD-neighbors and their interactions.

-*AD-brain seed-proteins*: proteins whose genes were differentially expressed in DNA array studies of brain tissue from AD patients [34].

-*AD-brain neighbors*: nodes selected as a consequence of adding experimentally validated interactions starting from AD seed-proteins.

-*AD-brain network*: network that includes AD seed-proteins, AD-neighbors and their interactions.

-*Disease-networks*: the term used to refer to the networks obtained from MS or AD patients that contain seed-proteins and their neighbors. It is important to note that we did not consider neighbors as newly proposed proteins implicated in the disease but rather, they were taken simply to capture the network context in which seed-proteins are located.

-*Giant component*: term used to refer the largest part of a network whose nodes are connected either directly or indirectly.

### Gene expression data

For the construction and analysis of the MS and AD networks, we selected seed proteins from previously published studies in blood [31,33] and brain [32,34].

### Network modeling

Starting from seed-proteins involved in either MS or AD, we obtained a PPI network through the interaction of these proteins with their direct neighbors. A general scheme of the approach adopted here is presented in Figure 1. The growth of each network was carried out using the STRING database [18] and the parameters used to generate the network in the STRING database were: active prediction method – experiments; confidence score – 0.7-high confidence; network depth – 2 (only direct neighbors); and an edge scaling factor of 80%. This configuration implies that only the experimental evidence of interactions with a high level of confidence were extracted from the database as valid links for each PPI network. A detailed description of each parameter can be found elsewhere [18]. We did not consider either the direction of each protein interaction or self-interactions. Network files in Pajek format (.net) of each network are available as additional files [see additional files 5, 6, 7, 8].

### Topological analysis and measurements of centrality

In order to characterize the disease networks (all of them undirected graphs) and assess the centrality of seed-proteins we applied the following measurements [25,35] using MATLAB (The Mathworks, MA, United States):

*-Degree (k)*: in an undirected graph, the degree of a vertex is the number of adjacent links. In this study, it represents the number of experimentally validated interactions (links) that connect one protein (node) to its neighbors.

-*Average degree (<k>)*: it represents the mean of all degree values of nodes in a network.

-*Clustering coefficient (<C>)*: is the average clustering coefficient of nodes where the clustering coefficient of a node *i *(*C*_*i*_) is the proportion of links between the nodes within the *i-*neighborhood divided by the number of links that could possibly exist between them.

-*Mean shortest path length (mspl)*: is the average of the steps (number of links) needed to connect every pair of nodes through their shortest path.

-*Diameter (D)*: is the longest among all shortest paths, i.e. the minimum number of links that separate the two most distant nodes in a network.

-*Betweenness centrality*: measures how often nodes occur on the shortest paths between other nodes. When combined with the degree, it is a key measure to assess the relevance of the location of nodes within a network (vertices within a graph).

### Gene Ontology

Gene symbol identities corresponding to the four different lists of seed-proteins were loaded into the ExPlainTm 2.3 Tool [36], where functional groups of Gene Ontology Biological Processes (GO-BP) were detected using a p-value threshold of 0.05 as the classification criteria and one as the minimal number of genes assigned to a group (i.e.: number of hits). A FDR multiple hypothesis test adjustment was further carried out using the Benjamini-Hochberg (BH) procedure [37] and taking the total number of GO-BP as those in which at least one protein of the seed-protein list is included.

### Statistical analysis

We used the Kolmogorov-Smirnov test to compare the distributions of degree and betweenness between seed-proteins and neighbors for each disease. The level of significance was set at *p *< 0.05.

## Abbreviations

PPI: protein-protein interaction; MS: Multiple Sclerosis; AD: Alzheimer Disease; CNS: Central nervous system; k: degree; <k>: averaged degree; <C>: clustering coefficient; D: diameter; mspl: mean shortest path length; GO: Gene Ontology.

## Authors' contributions

Study design: JG, SA, PV; Literature and Database search: JG, FE, IA; Network analysis: JG, FE, NV; Statistical analysis: JG, JS, NV; Analysis of results and writing the paper: JG, PV.

## Supplementary Material

Additional file 1 Table s1 ms-blood. Click here for file

Additional file 2 Table s2 ms-brain. Click here for file

Additional file 3 Table s3 ad-blood. Click here for file

Additional file 4 Table s4 ad-brain. Click here for file

Additional file 5 msblood. Click here for file

Additional file 6 msbrain. Click here for file

Additional file 7 adblood. Click here for file

Additional file 8 adbrain. Click here for file
